# An Embedded Multi-Agent Systems Based Industrial Wireless Sensor Network

**DOI:** 10.3390/s17092112

**Published:** 2017-09-14

**Authors:** Mohammed S. Taboun, Robert W. Brennan

**Affiliations:** Schulich School of Engineering, University of Calgary, Calgary, AB T2N 1N4, Canada; rbrennan@ucalgary.ca

**Keywords:** wireless sensor networks, multi-agent systems, embedded agents, distributed intelligent sensing and control

## Abstract

With the emergence of cyber-physical systems, there has been a growing interest in network-connected devices. One of the key requirements of a cyber-physical device is the ability to sense its environment. Wireless sensor networks are a widely-accepted solution for this requirement. In this study, an embedded multi-agent systems-managed wireless sensor network is presented. A novel architecture is proposed, along with a novel wireless sensor network architecture. Active and passive wireless sensor node types are defined, along with their communication protocols, and two application-specific examples are presented. A series of three experiments is conducted to evaluate the performance of the agent-embedded wireless sensor network.

## 1. Introduction

Recent advances in technology have led to the emergence of devices that connect to the Internet, in order to communicate with each other. This phenomena is known as the Internet of Things (IoT) and is revolutionizing daily life. The industrial application of these devices is known as cyber-physical systems; while a subset of IoT, cyber-physical systems, is composed of cyber-physical devices that have more focused requirements of IoT devices.

Wireless Sensor Networks (WSNs) are a widely-accepted solution to deal with the requirements and challenges of cyber-physical systems. WSNs are a large group of wireless sensor nodes, which communicate sensory data to a data acquisition system. Two key challenges, which are related to the reduction of power consumption of WSNs, are topology management and communication overhead reduction. Given the distributed nature of WSNs, multi-agent systems are a widely-accepted solution for managing WSNs.

In this paper, we propose an embedded multi-agent system to manage an industrial WSN. The WSN described in this study is targeted to the oil and gas industries; more specifically, oil and gas refineries along with storage tank and trailer monitoring applications. Some application-specific examples are provided in Section 6. After an outline of background information, an embedded agent architecture is proposed to manage and control the WSN. Two wireless sensor node types are presented: active and passive wireless sensor nodes. Two application specific examples are presented: a series of experiments is conducted to compare the effects of increasing the number of sensor nodes in a passive wireless sensor cluster, comparing a cluster of active wireless sensor nodes with passive wireless sensor nodes and, finally, the effect of a sensor node changing clusters.

## 2. Background

In this section, the background information is briefly examined. Cyber-physical systems are briefly described along with research trends in wireless sensor networks. Multi-agent systems as a solution for managing wireless sensor networks are also briefly outlined.

### 2.1. Cyber-Physical Systems

Cyber-physical systems are defined by [1] as “a new generation of systems with integrated computational and physical capabilities that can interact with humans through many new modalities.” In other words, cyber-physical systems are systems of hardware, with companion software, that are able to communicate with humans and other systems, as well as interact with their respective environments. This interaction is accomplished by sensing the environment (with sensors) and acting on the environment via some sort of actuation [2].

Cyber-physical systems have become a popular research area in the past decade. This research has presented several unique challenges in the context of designing secure and efficient cyber-physical systems. According to [3], these challenges arise when the computational framework is combined with a continuous physical system. Another challenge is also presented, in which cyber-physical systems must be autonomous. This is evident as many cyber-physical systems are tending to be designed to function without human intervention. An example is given with the self-driving car [4]. The third and final challenges presented in [3] are that most cyber-physical systems are constrained by environmental and operational limitations. Examples of these limitations are given as low computational power, battery life considerations, communication ranges and bandwidth.

A widely-accepted solution to contend with several of these challenges is the use of Wireless Sensor Networks (WSNs). WSNs are a network of small, low-powered wireless sensors. These sensors are usually battery powered and communicate through their own wireless sensor network protocol.

### 2.2. Industrial Wireless Sensor Networks

As previously mentioned, WSNs are a widely-accepted solution for some of the challenges in cyber-physical systems. A WSN is composed of wireless sensor nodes, which are small low-powered devices with limited processing and computing resources and are inexpensive compared to traditional sensors [5]. More specifically, a WSN sensing and monitoring system does not require the extensive electrical wiring infrastructure of a conventional sensing and motoring system. However, the trade-off can be lower speeds and higher latency due to the wireless interface. In recent years, industrial WSNs have become a popular area in research due to the advances in processing power of single-board micro-computers and the reduced power consumption of embedded devices.

**Figure 1:**
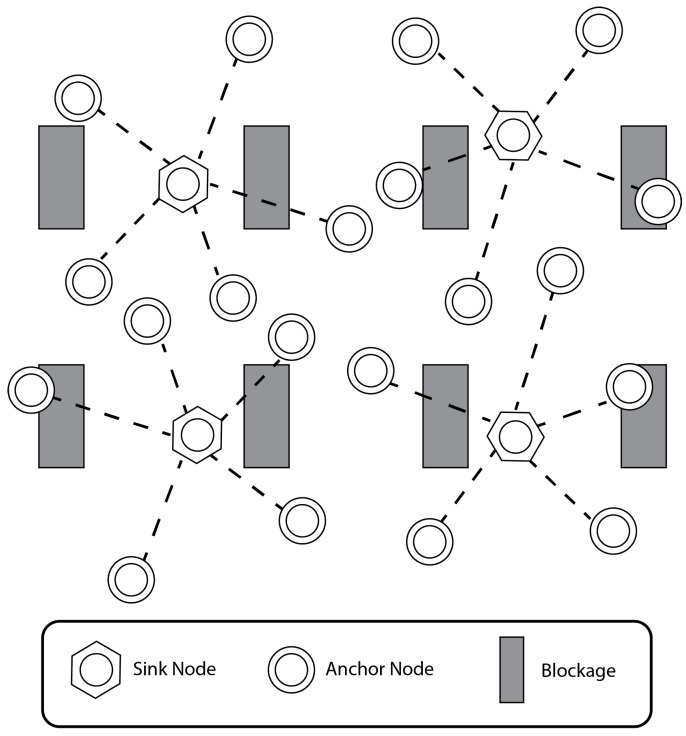
An industrial wireless sensor network with sink nodes and sensor nodes.

Two of the primary concerns in industrial wireless sensor networks are data routing and aggregation. When a large-scale industrial WSN processes data from many sensor nodes, a large bandwidth overhead is created. One of the most widely-accepted solutions that reduces this bandwidth overhead is clustering the WSN. This clustering process creates a hierarchical structure in the WSN, which allows for more streamlined data aggregation. The clustering process also creates two categories for classifying nodes within the WSN. These node types are sink nodes and regular sensor nodes, which are shown in Figure 1.

The cluster heads of the WSN’s respective clusters are made up of the previously-mentioned sink nodes. The sink nodes are responsible for aggregating data and transmitting this aggregated data from the WSN to the data acquisition centre (such as a SCADA system) or to the base station of the WSN. Due to the higher amount of transmissions required of the sink node when compared to the sensor nodes, the sink node is often a higher processing, fixed unit with respect to the sensor nodes. In other words, the sink node is a higher powered, wired unit with a fixed location.

The regular sensor nodes, on the other hand, make up most of the WSN. These sensor nodes collect sensory data and transmit these data to the sink node. The sensor nodes are therefore only required to have a very low powered processor and can usually be powered by a battery. In the case of networks where not all of the nodes are stationary, sensor nodes that have a fixed location are called anchor nodes, and nodes that do not have a fixed location are referred to as mobile nodes.

WSNs are distributed in their nature. There are several approaches to managing WSNs through distributed computing. One of the most widely-accepted solutions to manage WSNs is the use of multi-agent systems.

### 2.3. Static and Dynamic Clusters in Wireless Sensor Networks

In the previous section, clusters of wireless sensor nodes were introduced. An important factor when considering the design of wireless sensor networks is whether the clusters of wireless sensor nodes are static or dynamic. In a static cluster of sensor nodes, the sensor nodes are permanently assigned to a cluster and thus only perform tasks according to their respective cluster head. In a dynamic cluster however, the sensor nodes may change clusters, and their tasks and priorities may change depending on the cluster that they belong to at any given time [6].

### 2.4. Range Measurement Techniques in Wireless Sensor Networks

Range measurement techniques can be classified into five different techniques as described in [7]: Time of Arrival (ToA), Time Difference of Arrival (TDoA), Received Signal Strength (RSS), Angle of Arrival (AoA) and Frequency Difference of Arrival (FDoA). ToA estimates the distance between two neighbouring nodes by measuring the time the signal takes to travel between the two nodes. TDoA is similar to ToA in the sense that it utilizes the time taken to reach the destination. TDoA, however, measures the difference in arrival time of different types of signals sent at the same time to measure the distance from the source. RSS estimates the distance of the source sending the signal based on how strong the received signal is. AoA measures the angles of signals received from sources whose positions are known and uses these measurements to locate the position of the sensor node. Finally, FDoA measures the difference between received frequencies across two different antennas and uses the Doppler shift to calculate the distance from the source.

While time-based measurements such as ToA and TDoA provide a more accurate measurement of distance, they require a complex handshake interaction or multiple signal types respectively. AoA and FDoA require multiple and directional antennas. The advantage of RSS, as described in [7], is that sensor nodes do not need additional hardware or any additional communication overhead such as in the other techniques. For the previously-described reasons and due to the hardware constraints on the testing equipment, this study uses the RSS technique to estimate distance.

### 2.5. Agents in Wireless Sensor Networks

As mentioned previously, multi-agent systems are a widely-accepted solution for managing WSNs. In this section, key works in which multi-agent systems are used to manage wireless sensor networks are examined. It is, however, important to understand what is meant by the terms “agent” and “industrial agent.”

Although there is no unanimously agreed upon definition of a software agent, the most commonly-accepted definition of an agent is presented in [8]. This states that an agent in a computer system is situated in some environment and is capable of autonomous action in this environment to meet its delegated objectives. Agents are defined by their characteristics and behaviours. Agents, according to [9], are autonomous, responsive, proactive, goal-oriented, smart-behaving, social and able to learn.

Industrial agents, according to [9], share the features of an agent, but have some specific characteristics, as well. An industrial agent understands and efficiently handles the interface of low level industrial devices. Industrial agents also belong to an agent-based industrial application system within which hey act and communicate in a goal-oriented way. Industrial agents are subject to guidelines, codes of conduct, general laws and relevant directives from higher levels.

In [10], various multi-agent solutions for WSNs are examined. A multi-agent architecture that interconnects a wide range of heterogeneous devices that may possess various levels of resources is proposed in [11].

Tynan et al [12] proposed the development of intelligent sensor networks using multi-agent systems. This study had a simulation built, where the multi-agent system was developed using the Java Agent Development framework (JADE) in Java.

A multi-agent based application-oriented middle-ware was introduced in [13]. This middle-ware had a multi-agent management system that managed a distributed control system using IEC 61499 distributed control function blocks. This WSN middle-ware referred to a set of tools that reduced the complexity on lower level hardware systems for mobile object tracking. This middle-ware approach was illustrated using a case study in a factory mobile object tracking.

In [14], an interoperable WSN model using a multi-agent-based middleware was proposed. This model aimed to provide a generalized framework to address some critical issues in WSN paradigms such as interoperability, time synchronization, power management and distributed computation. The middleware proposed in this study allowed the authors to build a WSN system with efficient parallel processing capabilities and a higher reliability of system operations.

In [15], the “never miss an opportunity” scheme is proposed as a prototype for a WSN structure using multi-agent technology to provide agility to the WSN. This model used PB-MAC-based techniques to prioritize messages in order to wake sleeping sensor nodes and passively capture data during the occurrence of unpredicted events.

In [16], a multi-agent architecture is proposed to exploit the advantages of multi-agent systems modelling for WSN services, network topologies and sensor device architectures. This architecture was intended to facilitate the high-level design of sensor networks based on multi-agent systems. This model included agent-oriented modelling of WSN services and sensor device architectures. The authors expected this architecture to reduce the costs of developing complex autonomous systems and proposed validating this architecture as a future work.

Similarly, [17] proposed promoting the use of agent paradigms for the development of WSN applications. This study described the use of MAPS, the agent platform used in the study. The authors showed that the MAPs framework supported the development of applications in the context of WSNs. The requirements of a full-fledged agent-oriented methodology for the development of WSNs are also discussed.

In [18], multi-agent systems were used to simulate a large-scale WSN, agent protocols were used to facilitate the communication in the WSN. The aim of this study was to collect data using agents and to send them to a sink node. This simulation clustered the WSN using the widely popular LEACHtechnique. Another goal of this architecture was to have sensor nodes cooperate to eliminate redundant data. Successive simulations showed the ability of the agent-based WSN to reduce energy consumption and increase the rate of packet delivery in the WSN.

In [19], a schema for data collection based on multi-agent systems using mobile agents was proposed for collecting information according to an optimal reliable routing schema. This study presented successive simulations for large-scale WSNs based on the SINALGOsimulator and showed the performance of the architecture proposed in this study. The primary metrics of concern in this study were energy consumption and the rate of packet delivery.

In [20], a low-cost embedded agent architecture for crop monitoring and irrigation was proposed. This approached used the PANGEA [21] architecture to overcome the limitation of the low processing power and communication devices of the WSN. The intended outcome of this approach was the ability to merge heterogeneous environmental data from the WSN and initiate a response accordingly. In this study, a case-study in which farmers could monitor the crops through the wireless sensor network via the use of a television was presented.

Many studies of agent-based techniques in which the agents control and manage the WSN employ a middleware or have the agents deployed on a cloud-based system. This is due to the limited processing capability of wireless sensor node hardware. Recent advances in technology, especially in the field of single board computers such as the Raspberry Pi Zero [22], have made it feasible to embed the agents directly into the hardware. Many advantages of the distributed deployment of agents, such as network reliability and lower communication overhead, have been described in [9]. In this study, we apply the use of embedded multi-agent systems to manage and control real hardware, which paints a picture of what embedded agents used in the application of industrial WSNs may look like.

## 3. Previous Work

In this section, recent work directly related to this study is discussed. Initially, previous work on agent-based WSN is discussed, followed by the authors’ work on dynamic reconfiguration of WSN using multi-agent systems. The authors’ previous sink-node embedded multi-agent systems-managed WSN is then discussed. Finally, the authors’ application-specific example in oil and gas refineries is mentioned.

### 3.1. Agent-Based WSN

In [23], a multi-agent systems-based WSN was developed for mobile object tracking systems. This multi-agent system simulated a mobile object tracking system with thousands of sensor nodes. This wireless sensor network used Time Distance of Arrival (TDoA) to measure the distance between anchor nodes and mobile objects and then used two-dimensional trilateration to track the position of the mobile object. The multi-agent system was used to overcome challenges in industrial WSNs, such as signal blockage or power loss. The simulation was built using JADE and Java.

Although this multi-agent-based simulation used the distributed computing inherently incorporated into agent-based programming, the deployment of the agents was centralized. In other words, the agents for the simulation were all on the same computer. This type of deployment is common with many existing agent-based solutions, which, as previously mentioned, communicate to low level hardware through the use of cloud-based communication protocols.

### 3.2. Dynamic Reconfiguration in WSN for Mobile Object Tracking

In [24], a distributed approach to manage wireless sensor node tracking in a factory environment was proposed. The approach used a computationally-efficient distributed k-means algorithm, which divided up the WSN into Voronoi cells. In [25], static and dynamic clustering approaches are compared. The distributed approach in [24] is used as a base to compare static and dynamic clustering, using a simulation created with JADE. The experimental results in this paper corroborated the efficiency of static clusters versus the robustness of the dynamic clusters.

In [6], an experiment was conducted to compare a mobile object tracking WSN with static vs. dynamic clusters.The experiment was conducted with a simulation that was implemented in JADE. The experimental results show that while static clusters have a lower messaging overhead, dynamic clusters prove to have a higher reliability. In other words, in the simulation environment, dynamic clusters had a lower amount of missed signals.

### 3.3. Sink-Node Embedded Agents

In [26], we proposed a sink-node embedded agent WSN. This WSN had agents embedded only in the sink nodes. The agents were the task manager, the device manager and the sink node mediator. In this multi-agent system, the task manager was responsible for managing local task-oriented data, such as the data required for sensing tasks, and aggregating data. The device manager was responsible for managing local device tasks, such as cluster reconfiguration. This multi-agent system used a mediator architecture in which the device manager and task manager communicated via the sink node mediator agent.

### 3.4. WSN in Oil and Gas Refineries

In [26], an application-specific example demonstrating the plug an play properties of the sink node-embedded WSN was presented. In this example, a WSN was used to monitor an oil and gas refinery. The application demonstrated how clusters could be used to represent equipment, thus allowing intelligent, seamless introduction of new equipment to a facility. Similarly, decommissioned equipment could be removed without affecting the rest of the WSN, due to the hierarchical structure that resulted from clustering the WSN.

## 4. Multi-Agent Systems Embedded WSN

In this section, a novel multi-agent system is presented. The agents that are embedded in the sink nodes are discussed, followed by the agents in the sensor nodes. The sensor node states are described, and the WSN architecture is described.

### 4.1. Sink Node Embedded Agents

**Figure 2:**
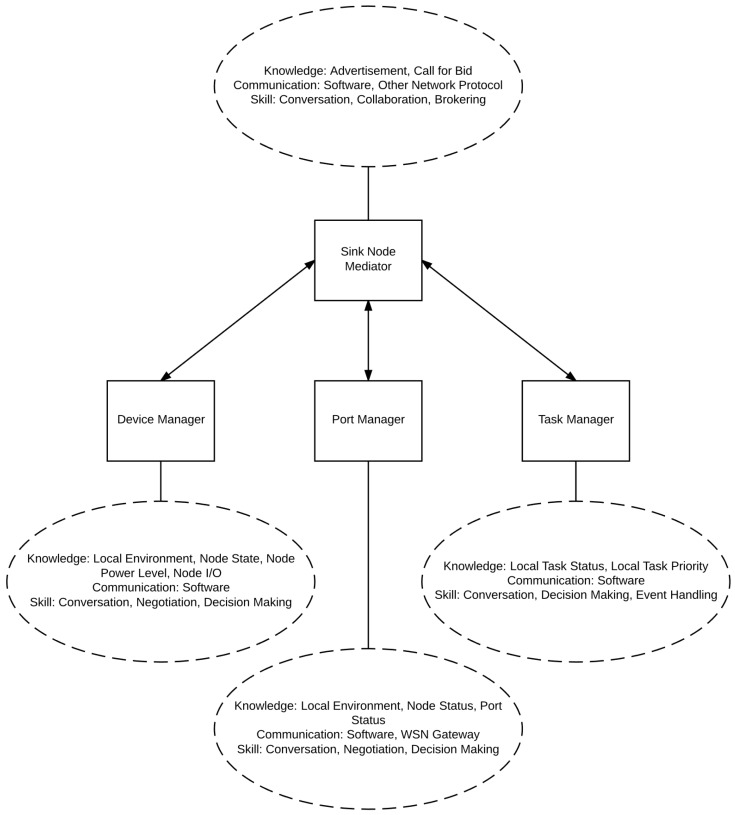
Sink-node embedded agents for agent-based WSN.

Built on the sink-node embedded agent system model initially described in [26], the sink node has several agents embedded in it. These agents are the task manager agent, the device manager agent, the port manager agent and, finally, the sink node mediator agent. These agents are described below and are shown in Figure 2.

In this architecture, the device manager has knowledge of sink nodes’ respective cluster (the local environment), the state of each node in the cluster (or nodes situated in the local environment), the residual power level of each sensor node and, finally, the I/O on the node. The device manager is only able to communicate through software APIs. The device manager has skills in conversation, negotiation and decision making.

The task manager has knowledge of each local task status, as well as the critical levels of local tasks. Similar to the device manager, it is only able to communicate through software APIs. The task manager has skill in data aggregation, integration, filtering, conversation, decision making and event handling.

The port manager is essential in managing data moving through the port through which the sink node communicates. This is necessary due to the elevated number of signals sent and received through the sink node when compared to a sensor node, combined with the port’s limited ability in sending or receiving a signal. In other words, the port manager should manage the port since a signal cannot be sent at the same time the device is listening for signals, nor can two signals be sent or received simultaneously. The port manager has knowledge of the local environment, the status of each node and the status of the WSN port. The port manager agent, like the previous two agents, can communicate through software APIs, but can also communicate through the WSN port. The port manager has skills in conversation negotiation and decision making.

Finally, the sink node mediator has knowledge of advertisements and calls for bids. It communicates through software APIs and other network protocols, for example through the Internet. The sink node mediator has skills in conversation, collaboration and brokering. The sink node mediator is responsible for mediating messages sent between agents located on a sink node, as well as communicating with agents on other sink nodes. The sink node mediator, however, does not mediate messages between sink nodes and regular sensor nodes local to the respective sink nodes.

### 4.2. Agent-Based Sensor Nodes

Similarly to the way agents are embedded into the sink nodes, the sensor nodes of the WSN have agents directly embedded into them. To clarify, it is important to first define “sensor node” and “sensor node agent”. The term “sensor node” refers to the hardware platform of the sensor. The term “sensor node agent” refers to the software component. In other words, “sensor node agent” refers to the embedded intelligence of the sensor node. In this architectures, there is one sensor node agent deployed on each sensor node.

The sensor node agent has a knowledge of the current cluster topology, or the local topology, neighbouring sink nodes, communication port status, critical task levels and task status. The sensor node agent is only to communicate with its respective sink node through the WSN protocol. The sensor node agent has skills in communication, data aggregation and decision making.

### 4.3. Node States for Dynamic Reconfiguration

**Figure 3:**
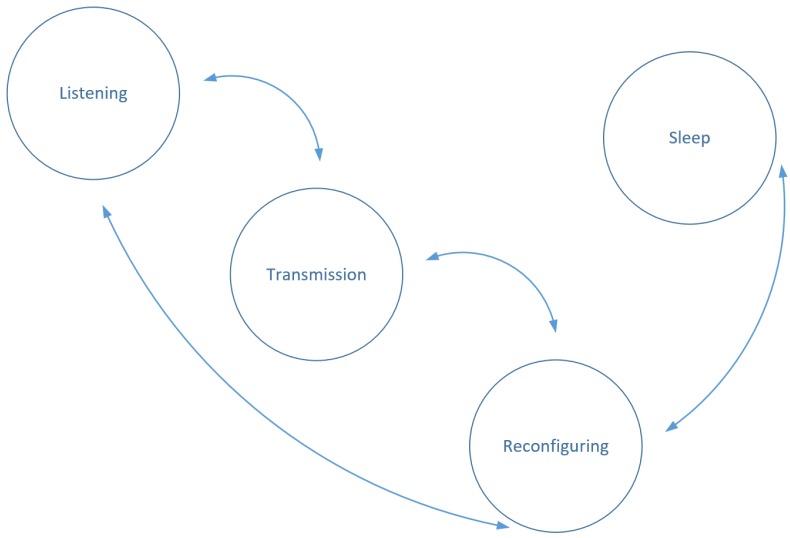
Sensor node states.

As previously mentioned, there may be a need for a sensor node to switch clusters. A reason for this can be a loss of communication with its’ respective sink node, due to blockage (see Figure 1) or a power outage for the sink node. Figure 3 shows the different anchor node states. The transmission state occurs when the sensor node is sending a message. Due to the resources required to send a transmission, this state is typically a high power consumption state. Listening is the state that the sensor would ideally spend most of its time in and, as a result, is its normal operating mode.

**Figure 4:**
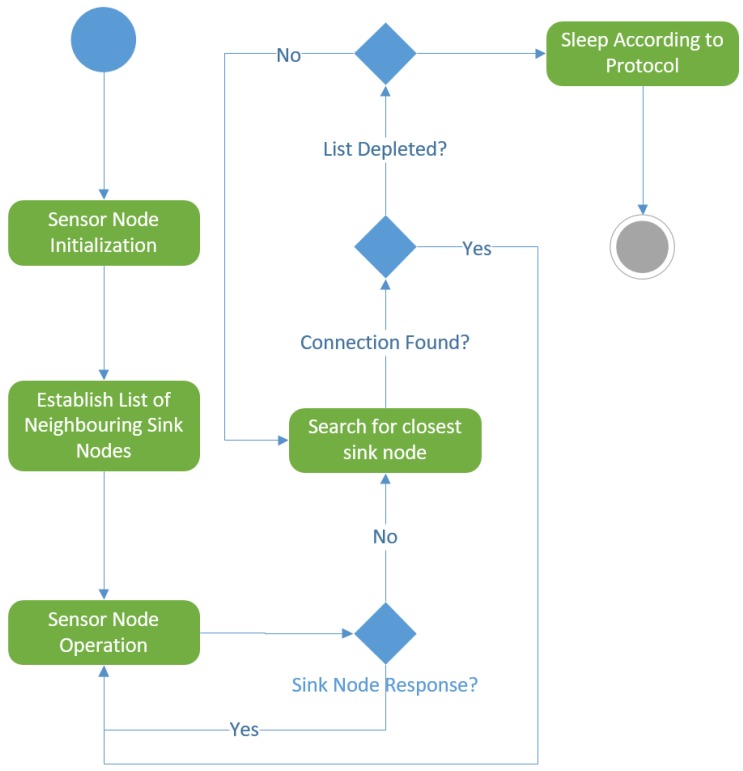
Sensor node setup and reconfiguration process.

The dynamic reconfiguration state occurs when a sensor node agent decides to change clusters. In other words, when a sensor node agent decides to change sink nodes, it enters the dynamic reconfiguration state. This state involves sending “pings” to its neighbouring sink nodes in a similar fashion to the channel scan technique and joining the closest possible sink node. Due to the amount of transmissions sent during this state, this is usually expected to be the node state with the highest consumption of power. If the sensor node agent is unable to find its’ respective sink node, it enters a sleep state, where it will lay dormant for a predetermined amount of time. When this time expires, the sensor node agent will enter the dynamic reconfiguration state again. This reconfiguration process, along with the initial node setup, is illustrated in Figure 4.

### 4.4. WSN Architecture

**Figure 5:**
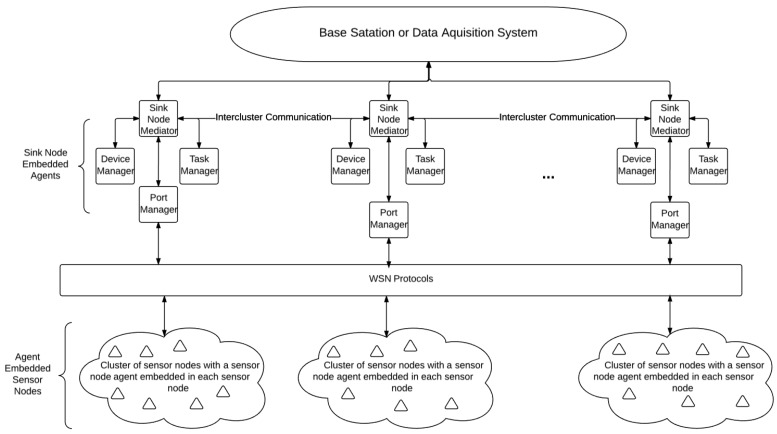
Wireless sensor network architecture.

As can be seen in Figure 5, sensor node agents are deployed on sensor nodes, which allows sensor nodes to communicate with the sink node of their respective clusters through the WSN communication protocols. The sink node embedded agents communicate with the data acquisition system and/or other sink nodes through other network protocols. Since the network architecture follows a clustered topology, scaling the network to more nodes is relatively simple. An industrial WSN can be composed of one, two or many sink nodes and one, two or many clusters of sensor nodes.

## 5. Active vs. Passive Sensor Nodes

In this section, we compare active and passive wireless sensor nodes. In order to illustrate the active and passive communication of sensory data between the sensor node and sink node, we must first define active and passive sensor nodes. For this study, we consider passive wireless sensor nodes to be sensor nodes that send sensor data on a given time interval, without a request from the sink node. Active wireless sensor nodes, on the other hand, will send sensory data to the sink node, only when the sink node sends a request first.

### 5.1. Active Wireless Sensor Nodes

**Figure 6:**
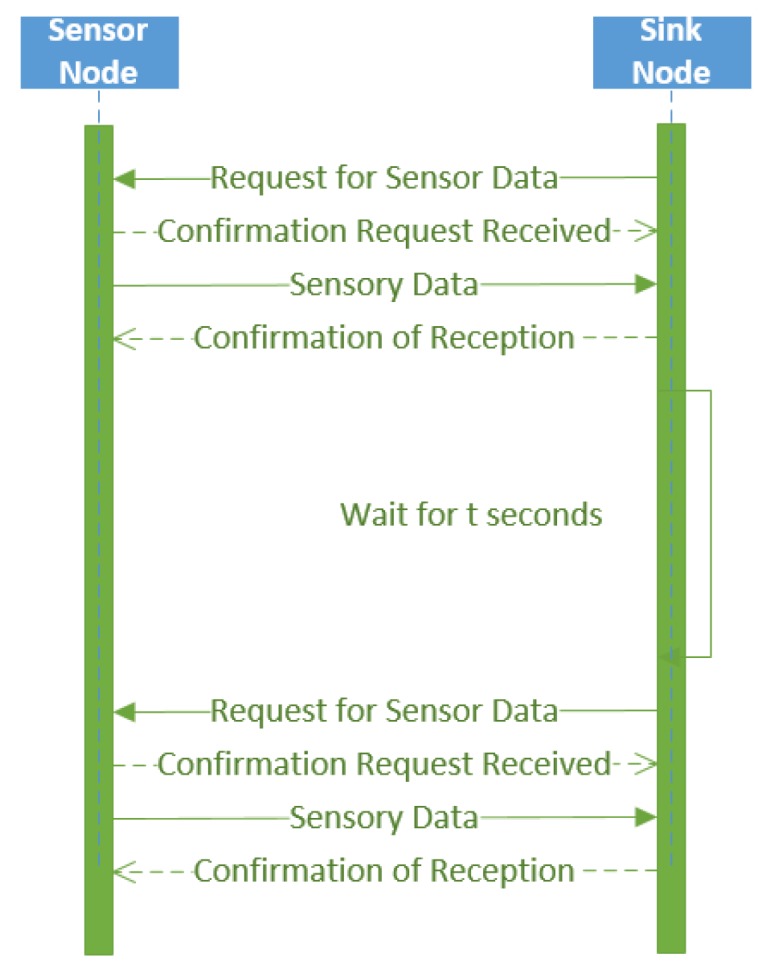
Active wireless sensor node communication with the sink node.

As previously mentioned, active sensor nodes will send sensory data only when requested by the sensor node. This process is initiated when the sink node sends a request. The sensor node confirms the request was received, then sends the sensory data. The sink node confirms the sensory data were received and then waits until it needs to request more sensory data (usually based on a sink node time interval). In other words, the active wireless sensor nodes use a query-based architecture to transmit sensory data upon request. This process is illustrated in Figure 6.

### 5.2. Passive Wireless Sensor Nodes

**Figure 7:**
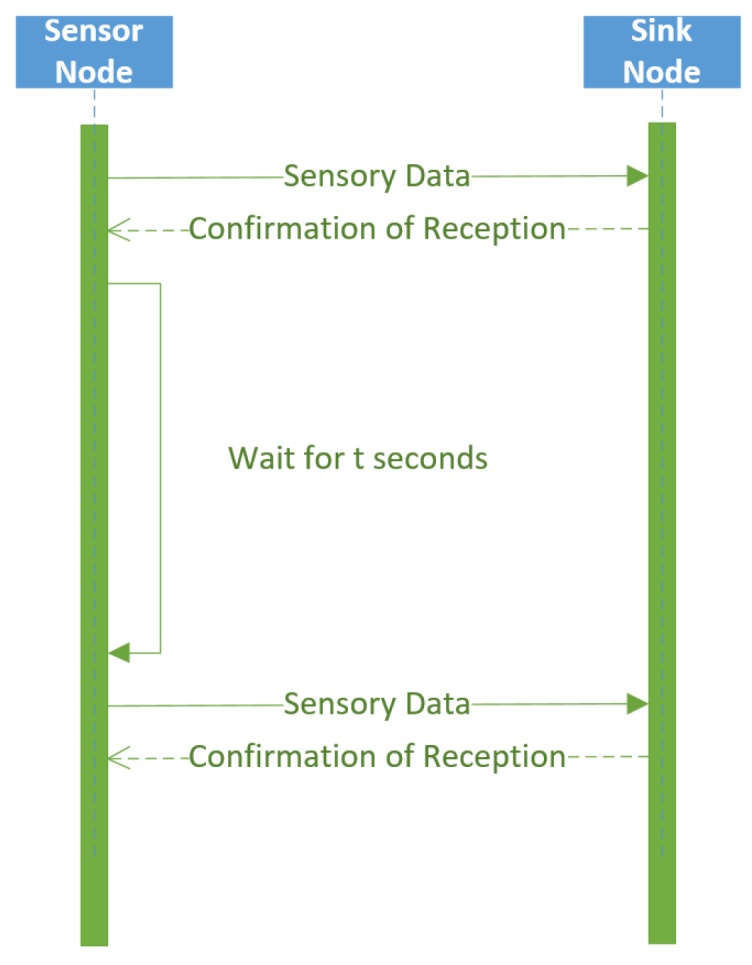
Passive wireless sensor node communication with the sink node.

Passive sensor nodes follow a more simplified process. The sensor node sends sensory data, and the sink node confirms there data were received. The sensor node waits for a predetermined time interval, then sends sensory data again. In other words, the passive sensor nodes use an advertisement architecture to passively transmit sensory data. This process is illustrated in Figure 7.

Due to the lower number of transmissions sent, the power consumption for each sensory data transmission should be lower when using a passive wireless sensor node. Active wireless sensor nodes, on the other hand, simplify cluster management: it is easier for the sink node to schedule received messages, which may result in a lower number of missed messages. In this study, we investigate the behaviour of WSNs with these two communication modes in the experiment sections.

## 6. Application Specific Example

In this section, we present two application-specific examples related to the oil and gas processing industries. The first example is for an oil and gas refinery, which was presented in [26]. The second example is a novel application in storage tank monitoring.

### 6.1. Agent-Based WSN for Oil and Gas Refineries

In [26], an application-specific example outlining the hierarchical clustering structure of a sink node embedded WSN in an oil and gas refinery is given. This example is still relevant when discussing a fully agent-embedded WSN. In this example, we consider the oil and gas refining process outlined in [27].

**Figure 8:**
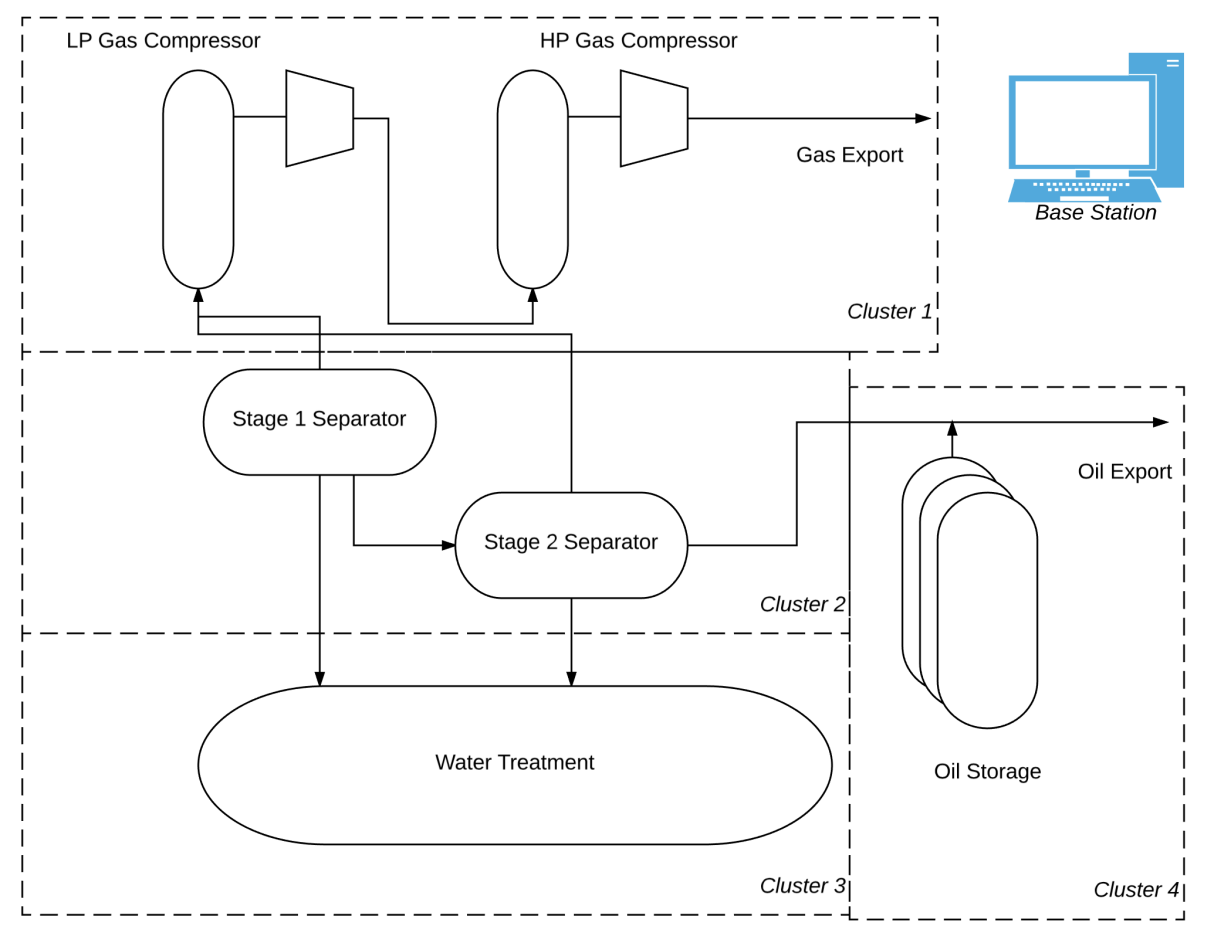
WSN for oil and gas refinery applications [26].

The basic refinery example starts with unprocessed oil (which consists of oil, gas and water) entering the Stage 1 separator. The natural gas that is separated from the Stage 1 separator and transported to the low pressure gas compressor. Meanwhile, leftover oil flows to the Stage 2 separator, where the remaining gas is separated from the oil. This gas is also sent to the low pressure gas compressor. All the while, water that is separated during the process is sent to the water treatment equipment. The refined oil is stored and/or exported after leaving Stage 2 separation. After leaving the low pressure gas compressor, the gas is sent to the high pressure gas compressor, where it is then exported through a gas pipeline. This process is illustrated in Figure 8.

Figure 8 also shows how cluster wireless sensor nodes can be used to monitor equipment. A cluster of wireless sensor nodes can be embedded into a group of machinery: for example Cluster 1 consists of wireless sensor nodes embedded in the low pressure and high pressure gas compressors. Similarly, a cluster of wireless sensor nodes can represent one piece of equipment, for example: Cluster 3 consists of wireless sensors embedded into the water treatment equipment.

### 6.2. WSN for Storage Tank Monitoring

A novel application-specific example is the use of agent-embedded WSN in a tank storage facility. Some industries make use of facilities with several tanks. Often, these tanks are attached to trailers and are moved from facility to facility. When monitoring the condition of the contents of the tank using a WSN, the sensors should be able to communicate with a base station or data acquisition system in the storage facility.

**Figure 9:**
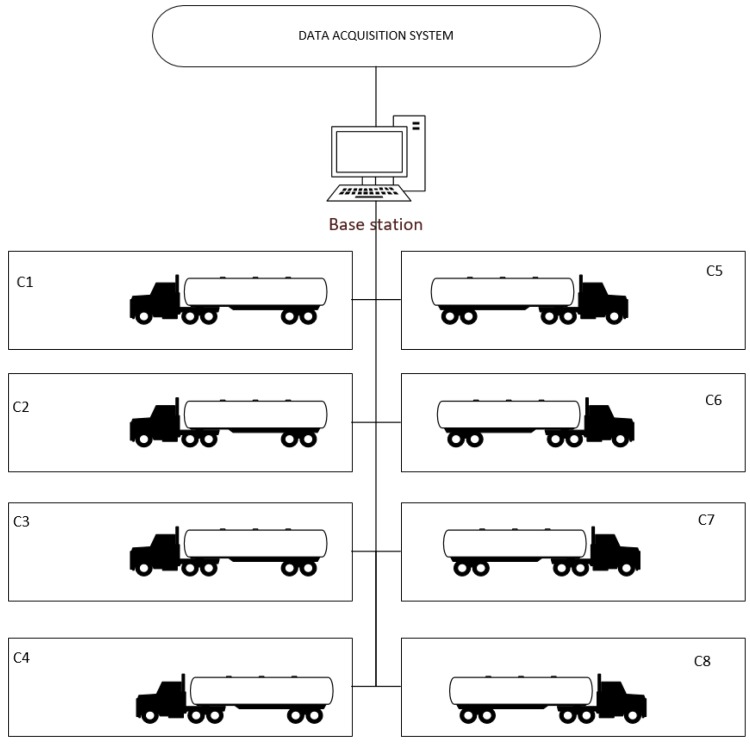
Example of storage tank facility with trailer attached tanks.

In this example, we consider the sink node to not be attached to the trailer. When a trailer leaves with a storage tank, the sensor nodes will go into a sleep state, conserving battery, or will communicate with a sink node that is on the towing vehicle. When a trailer enters a storage facility, the sensor nodes should join a cluster without human intervention. This allows the data acquisition system to seamlessly monitor all of the tanks in the facility. This example is illustrated in Figure 9.

One of the design requirements for the WSN in this application-specific example is the ability to add and remove sensor nodes from the wireless sensor networks. Sensor nodes are added when storage tanks arrive and similarly are removed when a storage tank leaves the facility. For this to occur, the topology of the wireless sensor network must consist of dynamic clusters. In the experiment section, the effect of dynamic clusters on the quality of service is investigated using a hardware test bed.

## 7. Experiments

In this section, the experiments and experimental methods are discussed. There are three experiments presented in this study. The first experiment compares two interval times for passive wireless sensor networks, as well as two different cluster sizes. The second experiment compares active and passive wireless sensor networks for different cluster sizes. The third experiment examines a sensor node reconfiguring its cluster. The metrics that are used to evaluate the performance of the WSN are discussed, followed by the hardware used for the experiment. The statistical design of the experiment is outlined, and the results are presented.

### 7.1. Metrics

The experiments are evaluated on three key metrics. The first metric is the lost signal ratio. The power consumption of the WSN is discussed along with the reconfiguration time.

#### 7.1.1. Lost Signal Ratio

In large-scale factory wireless sensor networks, one of the most critical metrics is the number of signals making it through to the data acquisition system. The Lost signal ratio (Ls) is defined as:

(1) $$L_{s}=\left(n_{sent}-n_{received}\right)/\left(n_{sent}\right)$$

where $n_{sent}$ is the total number of messages sent by sensor nodes in the WSN and $n_{received}$ is the total number of messages received by the sink nodes in the WSN.

While an acceptable lost signal ratio is application specific, typically a goal is to minimize the lost signal ratio. A lower lost signal ratio is the product of more of the signals sent by the sensor node read by the sink node. A higher lost signal ratio, on the other hand, is a product of the sink node not receiving the signals sent by the sensor nodes in its cluster. Lost signals result in lost information, which in turn degrades the quality of any monitoring and/or control processing. Therefore, a lower lost signal ratio contributes to a higher quality of service.

#### 7.1.2. Power Consumption

One of the most popular and heavily considered topics when considering the implementation of WSN is the battery power consumption. In industrial WSN, some or many sensors may be battery powered. In this experiment, power consumption is measured as opposed to battery power, as the sensors are all plugged into a power supply. We can define the total power consumption of the system as:

(2) $$P_{avg}=\left(\sum_{n=1}^{m}P_{tr,n}t_{tr,n}+P_{l,n}t_{l,n}+P_{r,n}t_{r,n}+P_{s,n}t_{s,n}\right)/m$$

where *n* is the current sensor node, *m* is the total number of sensor nodes, $P_{tr}$,$P_{l}$,$P_{r}$,$P_{s}$ are the power draw when a node is in its respective states, shown in Figure 1, and *t* represents the node’s time in each respective state.

It is important to note that only the power consumption for sensor nodes is of concern, as opposed to the power consumption of sink nodes. In a typical heterogeneous industrial WSN, the sensor nodes may or may not be battery powered, but the sink nodes are fixed units with an unlimited power supply. In this experiment, the power consumption of the sensor nodes is measured at the power source, through the use of a logging ammeter.

#### 7.1.3. Reconfiguration Time

The final metric, which is used in the third experiment, measures the reconfiguration time for the sensor node to change its cluster. This metric is important for several reasons. Firstly, the longer the sensor node spends in the reconfiguration state, the more power it uses. The second, and arguably the most important reason, is that while the sensor node is without a sink node, it cannot communicate critical sensory data. If the sensory data at the time are critical (for example, in an emergency), the lost signal ratio needs to be at a minimum. In this experiment, the reconfiguration time is measured by using the sensor node’s system clock and a data logger, which records each sensor node’s variable states, including the current sink node, in millisecond intervals.

### 7.2. Experimental Hardware and Software Implementation

**Figure 10:**
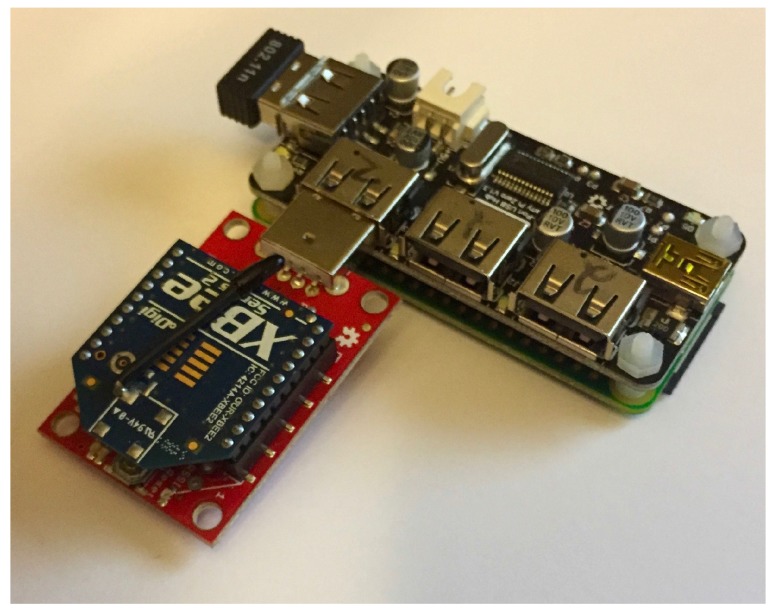
Photograph of Raspberry Pi Zero-based sensor node hardware.

In this study, the experiments are conducted on physical systems in order to capture realistic data as opposed to ideal-condition data, which simulated systems are prone to include. In order to understand the experimental results, it is important to understand the hardware configuration of the WSN. As previously mentioned, the sensor nodes are connected to a common power source, and a logging ammeter was used to measure the current draw. The sensor nodes are composed of two pieces of hardware. The hardware platform in which the sensor node agents are embedded on are a Raspberry Pi Zero v1.3 [22]. These Raspberry Pi Zeros are connected to their WSN components, which are ZigBee XBee nodes [28], using a standard XBee Explorer adapter. The sensor nodes also include a standard WiFi adapter; however, these adapters are only used for agent deployment and not in experimentation. A photograph of a sensor node can be seen in Figure 10.

**Figure 11:**
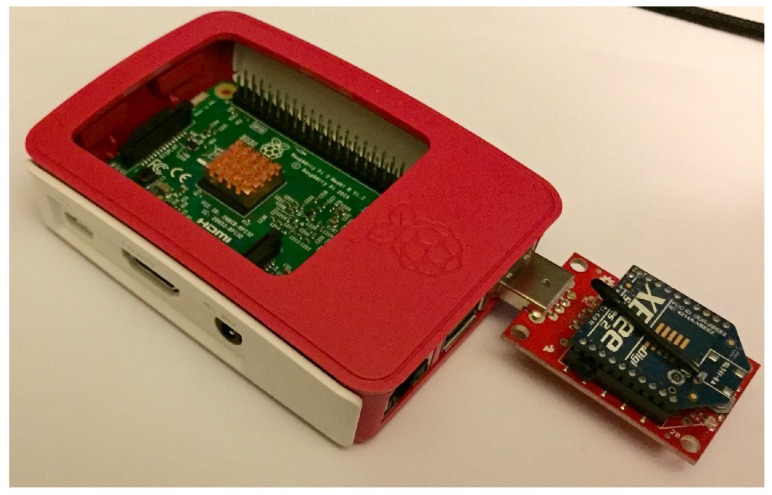
Photograph of Raspberry Pi 3-based sink node hardware.

The sink nodes use a Raspberry Pi 3 [29] as their hardware platform for intelligence. The Raspberry Pi 3 has a more powerful processor than the Raspberry Pi Zero and is capable of running many software agents at once. Like the sensor nodes, the sink nodes have ZigBee XBee nodes in which they communicate to their respective cluster of sensor nodes. A photograph of a sink node can be seen in Figure 11.

One of the key reasons for choosing Raspberry Pi is its ability to run Java programs natively. The XBee ZigBee API supports Java as its official language. Thus, the multi-agent system was implemented in the Java Agent Development Framework (JADE) [30]. The Raspberry Pi Zero and Raspberry Pi 3 run a Linux operating system (headless), and the JADE agents are deployed accordingly.

In this experiment, we are concerned with resource usage and quality of service in the clusters. In order to effectively compare factors, the types of messages in communication exchanges need to be consistent. As a result, the sensor nodes do not send actual sensor readings, but generic messages that follow the format: (SENSORID,MESSAGENUMBER,EXCEPTIONCODE). This allowed us to provide an objective comparison of the loading on the clusters.

### 7.3. Statistical Design

In the first experiment, we are investigating the effect of two factors on the lost signal ratio and the power consumption of a wireless sensor network cluster. The first factor is the number of sensor nodes in a cluster. When increasing the number of sensor nodes, the sink node requires more bandwidth, so we expect to see an increase in lost signal ratio. In this experiment, this factor has six levels, the cluster size ranging from 5–10 sensor nodes.

The second factor is the time between sent signals. In a WSN with passive sensor nodes, the sensor nodes send signals based on a factor internal to the sensor node. In this experiment, the sensor nodes are inspected based on a time frequency. This factor has two levels: 5 s and 10 s.

Given the previously introduced factors, a statistical scheme for experimentation can be devised. There are 2 factors, 1 of which is 6 levels and 1 of which is 2 levels. With a full factorial design outlined according to [31], we will have the following design:

(3) $$r_{base}=\left(6^{1}\right)\left(2^{1}\right)$$

where $r_{base}$ is the number of base runs. In the first experiment, there are 12 base runs. With 5 replications of these base runs, there are 60 runs required.

In the second experiment, we are investigating the effect of two factors, as well. The first factor is the effect of cluster size on a WSN cluster, similar to the first experiment. Using some of the data from the first experiment as a control group, the second factor compares the active and passive communication protocols discussed in Section 5. With the same number of factors, the same number of base runs is required as in the first experiment. Thus, with 5 replications, there are 60 runs altogether for the second experiment.

In the third experiment, we look at the dynamic cluster behaviour’s effect on the lost signal ratio, the power consumption of the sensor nodes and the reconfiguration time as opposed to static clusters. The effect of increasing cluster size with these two sensor node configurations is also examined. In this experiment, a sensor node switches back and forth between two clusters every 100 messages. The two clusters range in size from 5–10 sensor nodes. Similar to the first two experiments, there are 12 base runs and 5 replications. Thus, the third experiment also has 60 runs.

## 8. Experimental Results

In this section, the experimental results are discussed. As previously mentioned, there were three experiments conducted. First, a comparison of 5 s and 10 s transmission interval times for 6–10 sensor nodes is discussed. Next, active and passive wireless sensor nodes for a cluster of 6–10 sensor nodes is discussed. Finally, the effect of a cluster changing sink nodes is discussed.

### 8.1. Comparing Interval Times for Passive Wireless Sensor Nodes

In the first series of experiments, passive wireless sensor nodes with different interval times are compared. First, the effect of cluster size on sensor nodes with 5 s and 10 s intervals is presented. Then, the difference in the lost signal ratio and power consumption of the two interval times is presented.

#### 8.1.1. Effect of Cluster Size on Passive Wireless Sensor Nodes

**Figure 12:**
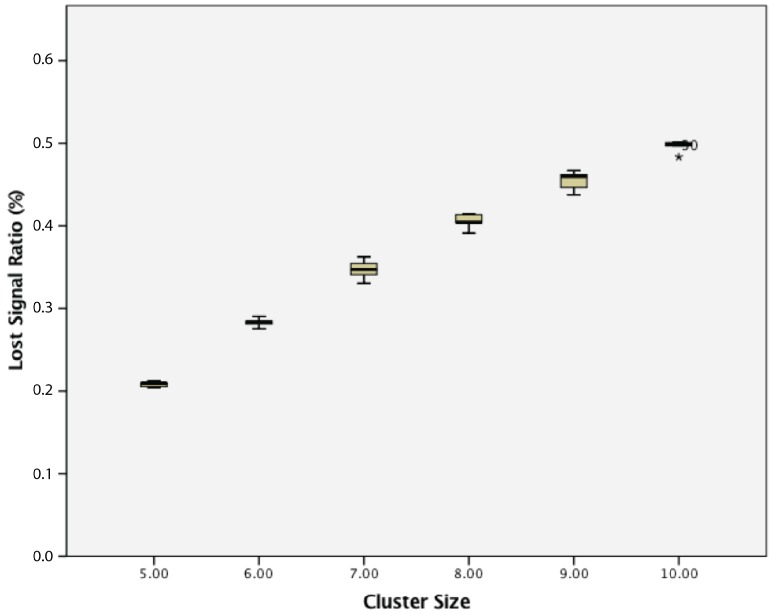
Lost signal ratio of cluster of 5–10 passive wireless sensor nodes with a 5 s transmission interval.

When considering the lost signal ratio, for a cluster of passive wireless sensor nodes with 5 s transmission intervals, there was a statistically-significant difference between groups as determined by one-way ANOVA (F(5,24) = 718.726, *p* = 0.000). A Tukey post hoc test revealed that the lost signal ratio became statistically significantly higher whenever an extra sensor node was added, as summarized in Figure 12.

**Figure 13:**
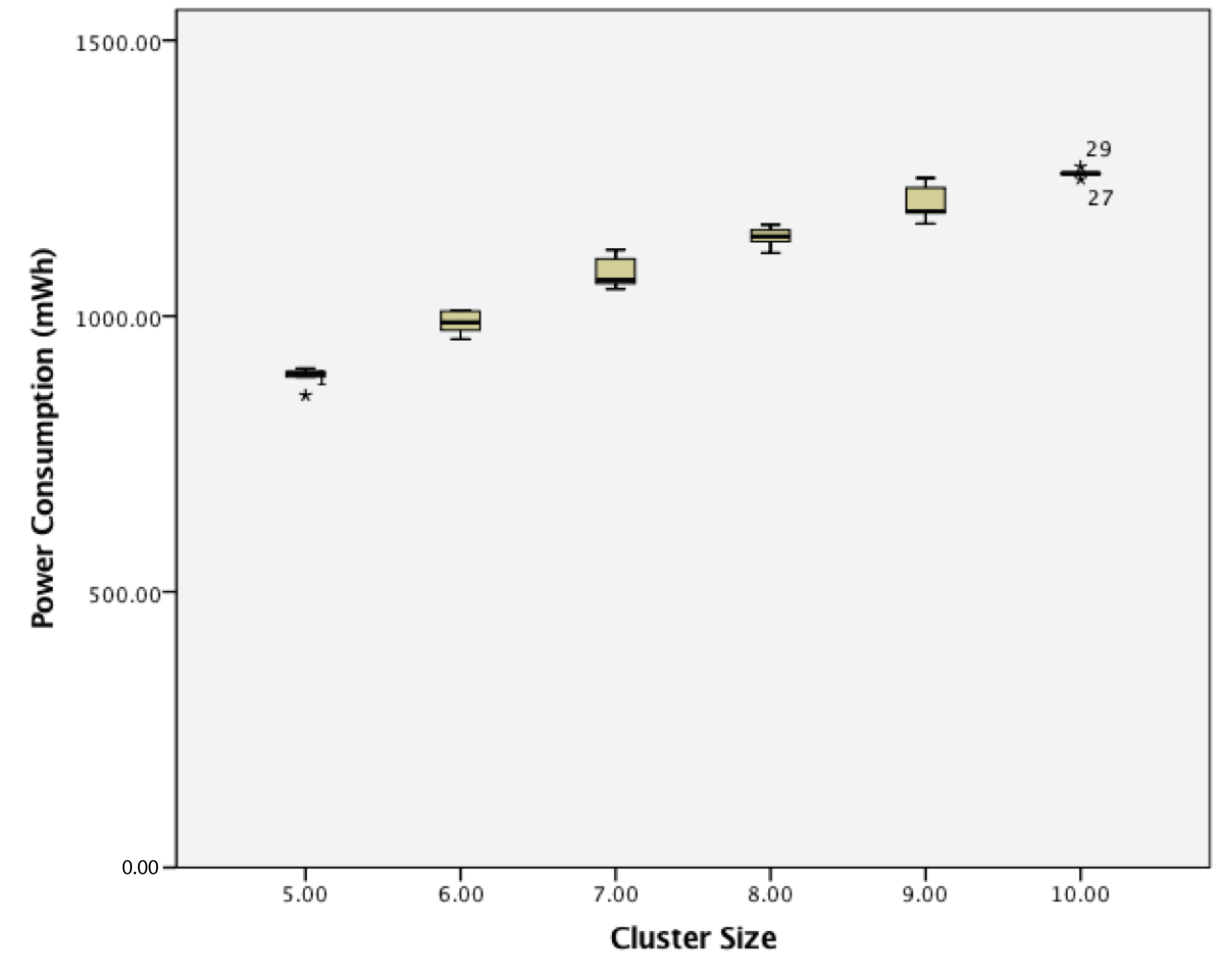
Power consumption of cluster of 5–10 passive wireless sensor nodes with a 5 s transmission interval.

For the power consumption for a cluster of passive wireless sensor nodes with 5 s transmission intervals, there was a statistically-significant difference between groups as determined by one-way ANOVA (F(5,24) = 165.675, *p* = 0.000). A Tukey post hoc test revealed that the power consumption became statistically significantly higher whenever an extra sensor node was added, as summarized in Figure 13.

**Figure 14:**
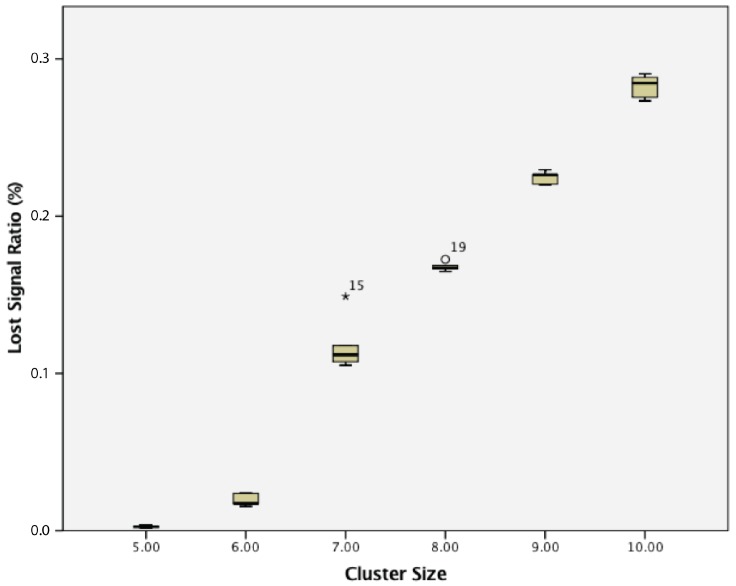
Lost signal ratio for clusters of 5–10 passive wireless sensor nodes with a 10 s transmission interval.

For the lost signal ratio for a cluster of passive wireless sensor nodes with 10 s transmission intervals, there was a statistically-significant difference between groups as determined by one-way ANOVA (F(5,24) = 887.512, *p* = 0.000). A Tukey post hoc test revealed that the lost signal ratio became statistically significantly higher whenever an extra sensor node was added, as summarized in Figure 14.

**Figure 15:**
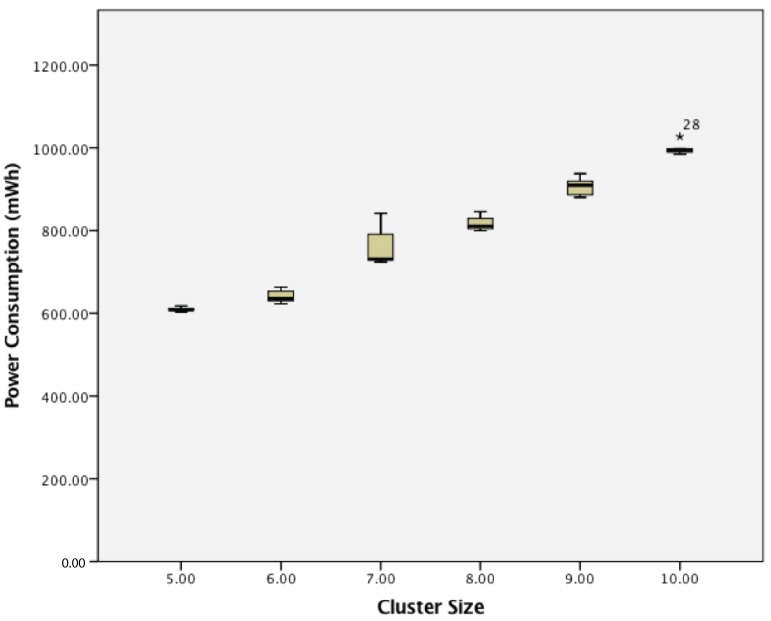
Power consumption of a cluster of 5–10 passive wireless sensor nodes with a 10 s transmission interval.

For the power consumption for a cluster of passive wireless sensor nodes with 10 s transmission intervals, there was a statistically-significant difference between groups as determined by one-way ANOVA (F(5,24) = 163.934, *p* = 0.000). A Tukey post hoc test revealed that the power consumption became statistically significantly higher whenever an extra sensor node was added, as summarized in Figure 15.

#### 8.1.2. The Effect of Increasing Passive Wireless Sensor Node Intervals

**Figure 16:**
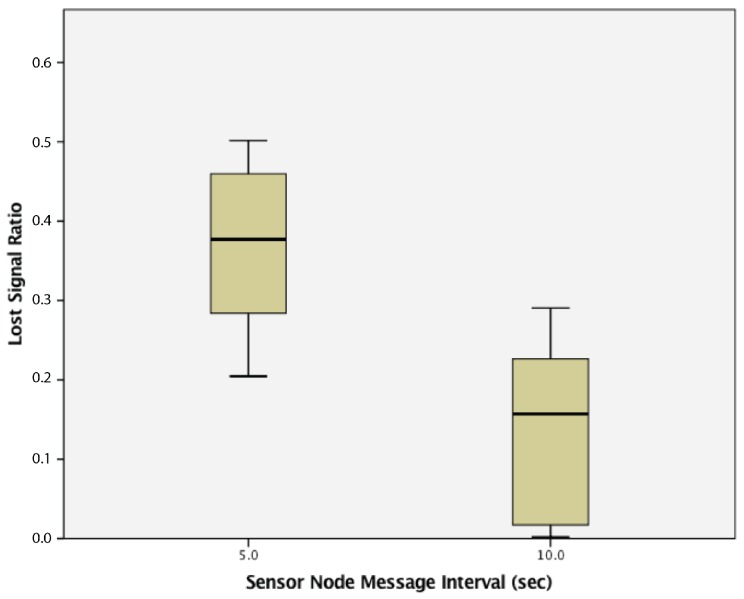
Lost signal ratio of clusters of passive wireless sensor nodes with 5 s vs. 10 s transmission intervals.

This study found that WSN clusters run with a 10 s transmission interval had statistically significantly lower lost signal ratios (0.136 ± 0.104) compared to WSN clusters run with a 5 s transmission interval (0.366 ± 0.101), t(58) = 8.714, *p* = 0.000. The results are summarized in Figure 16.

**Figure 17:**
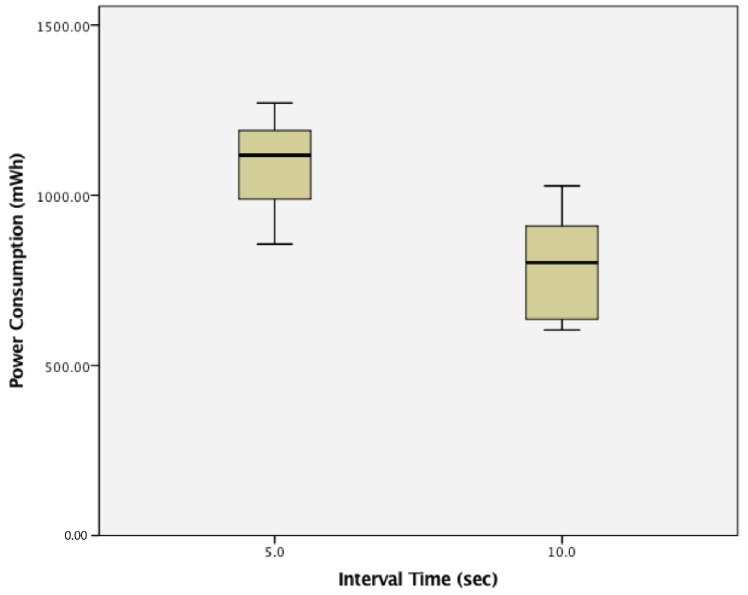
Power consumption of clusters of passive wireless sensor nodes with 5 s vs. 10 s transmission intervals.

This study found that WSN clusters that have a 10 s transmission interval had statistically significantly lower power consumption (789.7161 ± 141.828) when compared to clusters run with a 5 s transmission interval (1094.15 ± 130.137), as well, t(58) = 8.663, *p*= 0.000. The results are summarized in Figure 17.

### 8.2. Comparing Active and Passive Wireless Sensor Nodes

As previously mentioned, the second experiment compares active and passive wireless sensor nodes. First, the effect of cluster size and lost signal ratio are looked at, along with the difference between the means of the active and passive wireless sensor nodes. After that, the effect on power consumption is examined.

#### 8.2.1. The Effect of Cluster Size on Active and Passive Wireless Sensor Nodes

**Figure 18:**
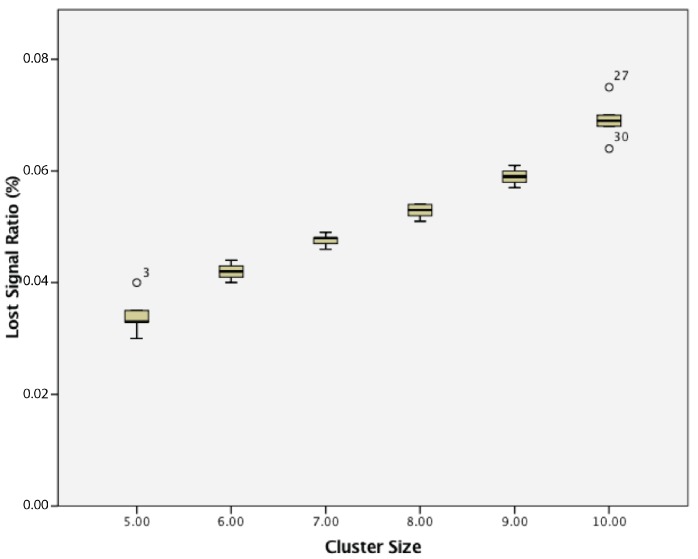
The effect of cluster size on the lost signal ratio of a cluster of active wireless sensor networks.

When considering the lost signal ratio for a cluster of active wireless sensor nodes, there was a statistically significant difference between groups as determined by one-way ANOVA (F(5,24) = 237.482, *p* = 0.000). A Tukey post hoc test revealed that the lost signal ratio became statistically significantly higher whenever an extra sensor node was added, as summarized in Figure 18.

**Figure 19:**
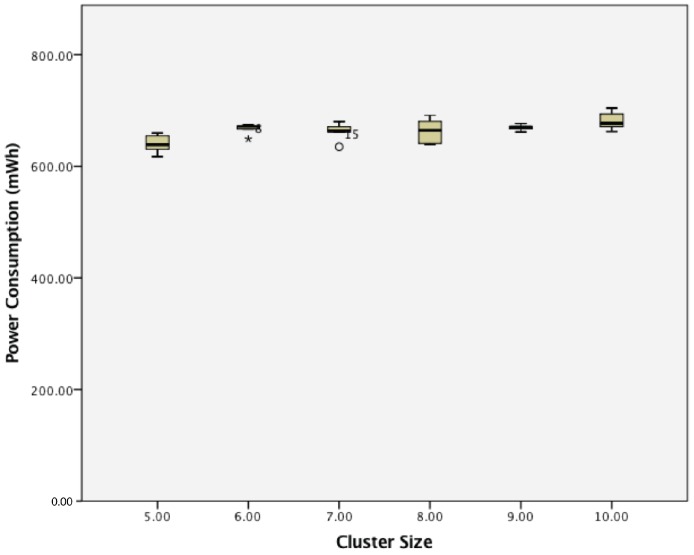
The effect of cluster size on the power consumption of a cluster of active wireless sensor networks.

When considering power consumption, for a cluster of active wireless sensor nodes, there was a statistically-significant difference between groups as determined by one-way ANOVA (F(5,24) = 3.569, *p* = 0.015). A Tukey post hoc test revealed that the power consumption became statistically significantly higher whenever an extra sensor node was added, as summarized in Figure 19.

#### 8.2.2. Active vs. Passive Wireless Sensor Nodes

**Figure 20:**
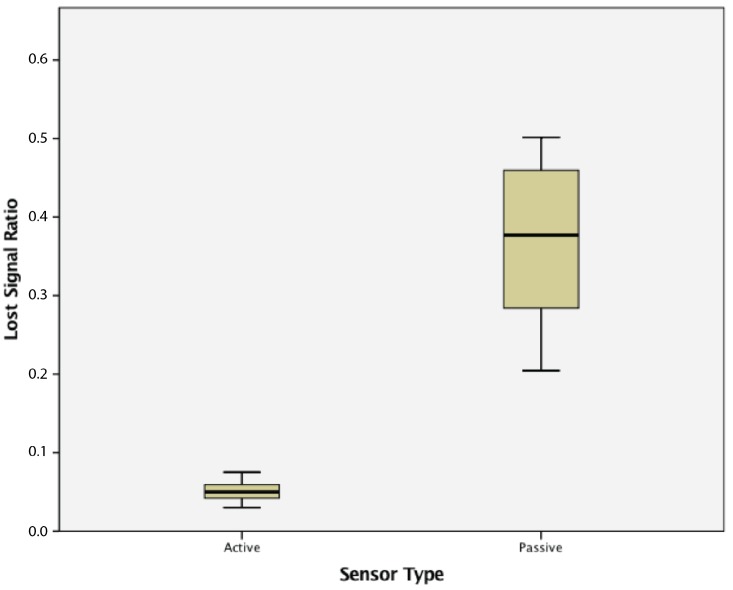
Lost signal ratio in a cluster of active vs. passive wireless sensor nodes.

This study found that WSN clusters of active wireless sensor nodes had statistically significantly lower lost signal ratios (0.0508 ± 0.1177) compared to clusters of passive wireless sensor nodes (0.366 ± 0.101), t(58) = 17.010, *p* = 0.000. The results are summarized in Figure 20.

**Figure 21:**
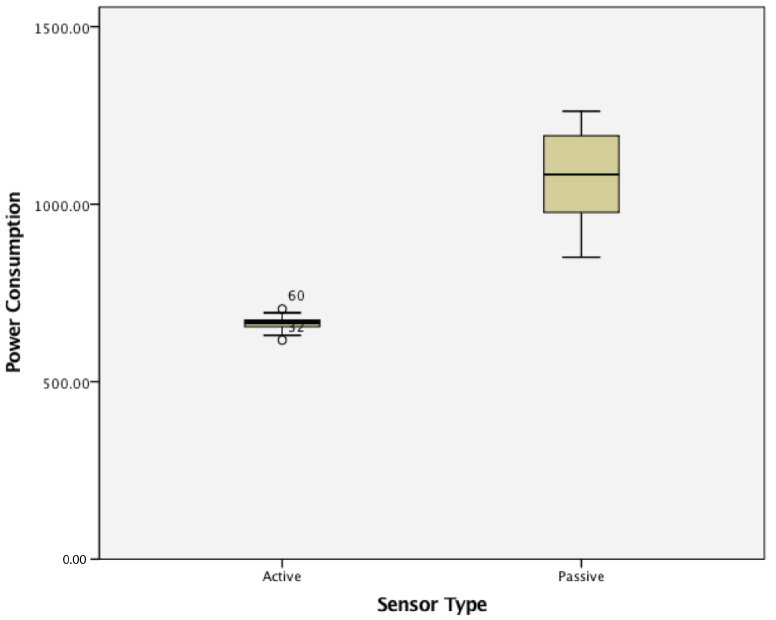
Power consumption in a cluster of active vs. passive wireless sensor nodes.

This study found that WSN clusters of active wireless sensor nodes had statistically significantly lower power consumption (663.92 ± 19.33) compared to clusters of passive wireless sensor nodes (1075.69 ± 19.33), t(58) = 16.726, *p* = 0.000. The results are summarized in Figure 21.

### 8.3. Effect of Sensor Node Cluster Reconfiguration

#### 8.3.1. Effect of Increasing Cluster Size

**Figure 22:**
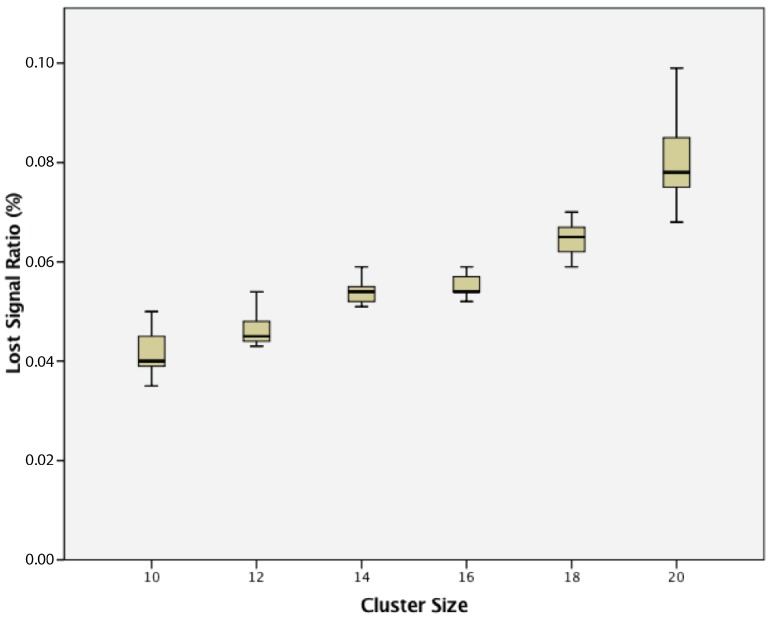
Lost signal ratio of clusters of dynamic wireless sensor nodes.

For a cluster of dynamic wireless sensor nodes, there was a statistically-significant difference between groups as determined by one-way ANOVA (F(5,24) = 25.821, *p* = 0.000). A Tukey post hoc test revealed that the lost signal ratio became statistically significantly higher whenever an extra sensor node was added, as summarized in Figure 22.

**Figure 23:**
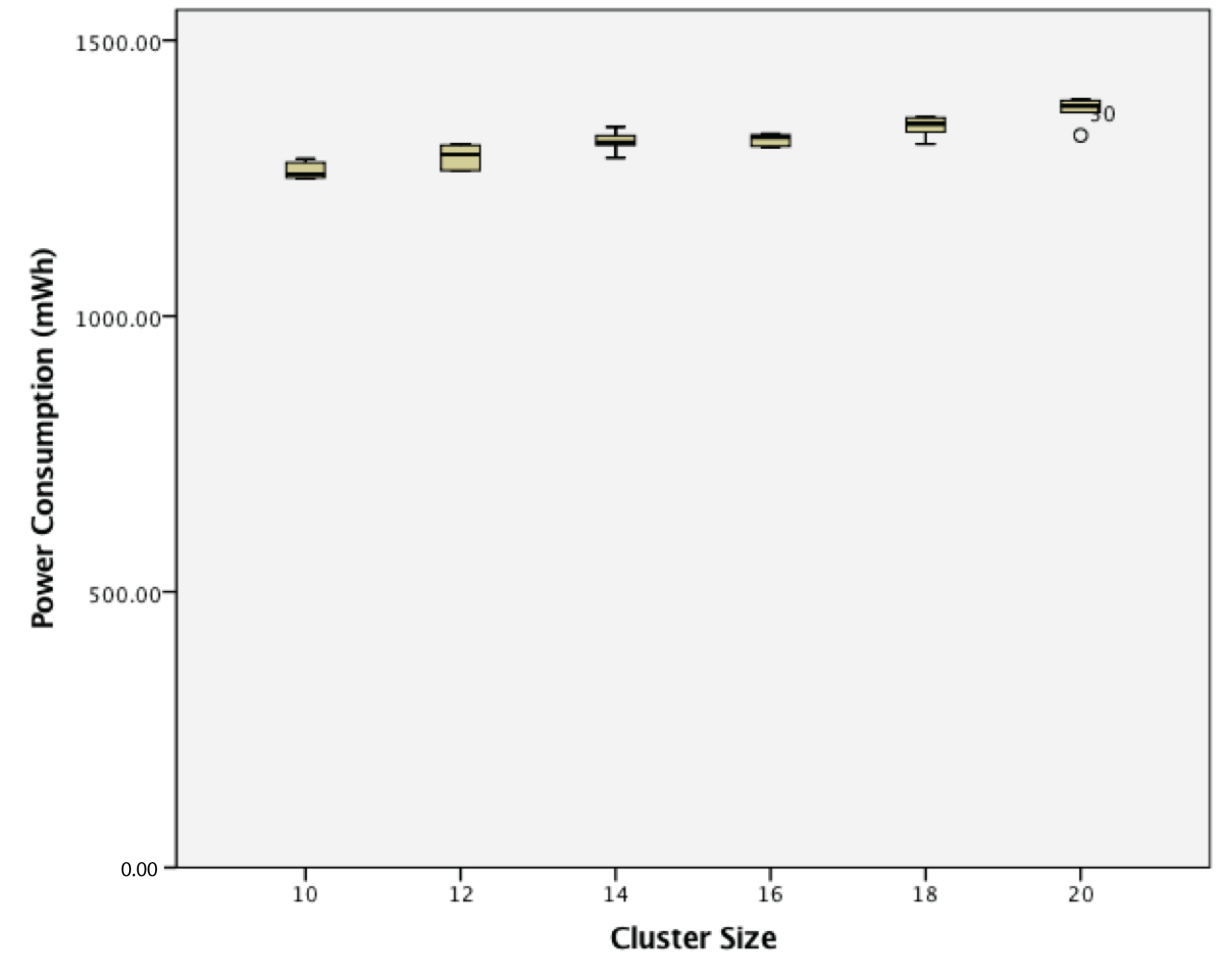
Power consumption of dynamic wireless sensor nodes.

When considering power consumption, for a cluster of dynamic wireless sensor nodes, there was also a statistically-significant difference between groups as determined by one-way ANOVA (F(5,24) = 10.145, *p* = 0.000). A Tukey post hoc test revealed that the power consumption became statistically significantly higher whenever an extra sensor node was added. These results are shown in Figure 23.

**Figure 24:**
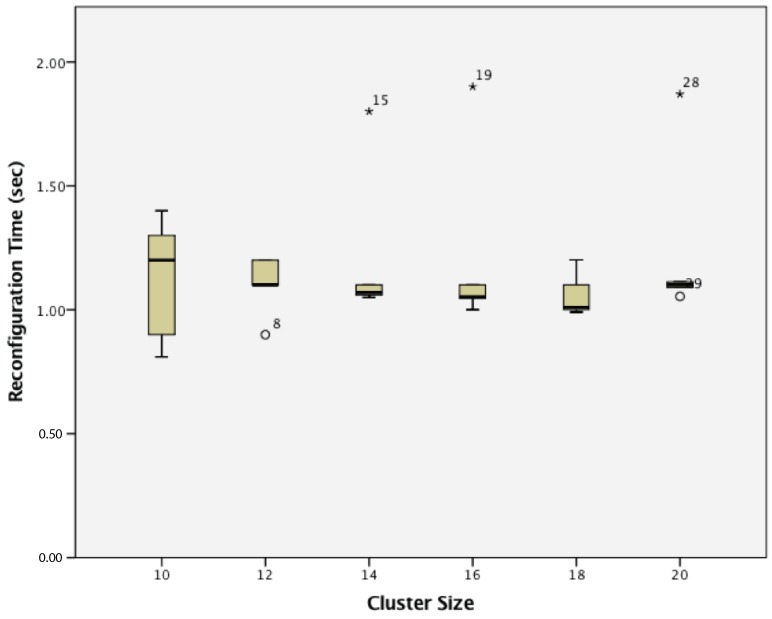
Reconfiguration time of dynamic wireless sensor nodes.

When considering reconfiguration time, there is no statistical-significant difference between groups. This can be seen in Figure 24.

#### 8.3.2. Static vs. Dynamic Clusters

**Figure 25:**
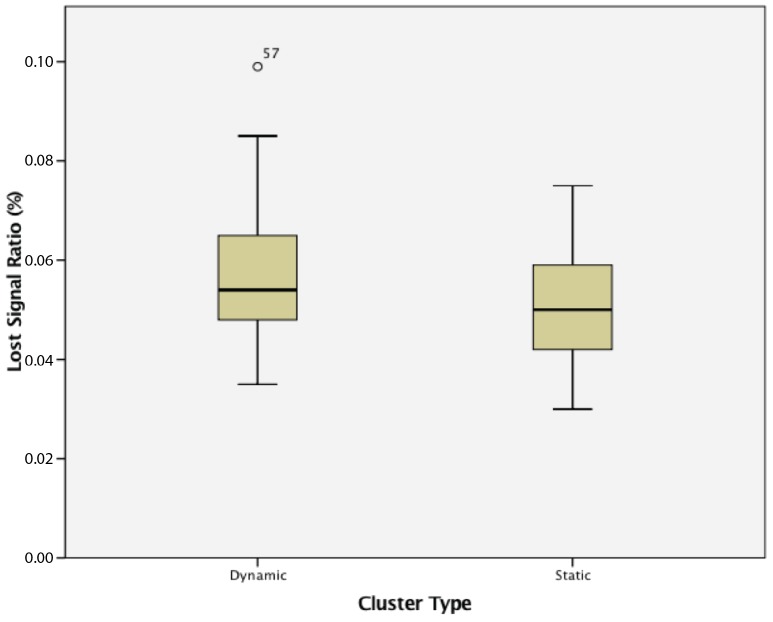
Lost signal ratio of static and dynamic wireless sensor nodes.

**Figure 26:**
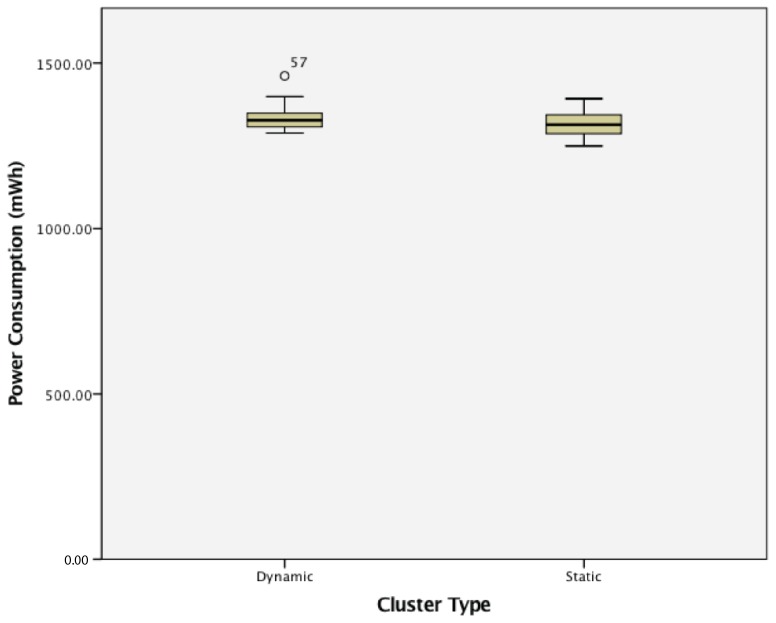
Power consumption of static and dynamic wireless sensor nodes.

This study found that WSN clusters of dynamic wireless sensor nodes were not significantly different when compared to static WSN both in terms of lost signal ratio and in terms of power consumption. These results are shown in Figure 25 and Figure 26.

## 9. Discussion

In the first experiment, sensor node clusters with a message interval of 5 s were compared with sensor node clusters with an interval of 10 s. The experiment found that for a cluster of passive wireless sensor nodes, increasing the number of nodes in the cluster resulted in an increase of the lost signal ratio, as well as an increase of power consumption per sensor node. The first experiment also found that increasing the interval between messages sent generally decreases both the lost signal ratio, as well as the power consumption of the WSN cluster. The effects of increasing the sensor nodes can be expected, due the much higher amount of bandwidth the sink node must receive. The increase in power consumption, however, can be linked to the higher amount of time spent in the transmission state as the anchor nodes try to send the same message multiple times before moving on to the next message, as per the ZigBee protocol.

In the second experiment, the increase in sensor nodes per cluster had less of an effect on the active system. While there was still a significant increase in both lost signal ratio and power consumption, the active wireless sensor networks tend to be less effected by the increase in cluster size. When compared to the cluster of passive wireless sensor nodes, the active sensor node clusters had a statistically significantly lower lost signal ratio and lower power consumption. These results show that the active wireless sensor nodes, which allow for ease of reconfigurability with the embedded multi-agent system, are also less prone to a high lost signal ratio while consuming less power.

Finally, the third experiment compared a static cluster with a dynamic cluster. The results show that while an increase in cluster size increases both the lost signal ratio and power consumption of the dynamic network, it does not affect the reconfiguration time. When comparing the differences between a static cluster of active wireless sensor nodes, there is no statistically-significant difference in lost signal ratio or the power consumption of the different groups. This demonstrates the WSNs’ capabilities when adding and removing wireless sensor nodes, which is essential to the reconfigurability of the industrial WSNs in the application-specific examples.

These experiments paint a picture of the effects of different factors on a wireless sensor network. When considering passive wireless sensor nodes, increasing the size of each cluster results in a lower quality of service, along with an increased power consumption for each sensor node. While the power consumption for a passive wireless sensor node on its own should be lower than that of an active sensor node due to the lower amount of transmissions required, a cluster of passive sensor nodes typically uses more power than a cluster of active wireless sensor nodes. This may be due to hardware protocols of the hardware platform on which the sensor node agents are deployed. When comparing static and dynamic clusters of sensor nodes, dynamic clusters have lower lost signal ratios, corroborating the simulation results in [6]. The experiments in this study show the types of results to expect when implementing embedded agents on a low-powered hardware platform as opposed to simulations.

## 10. Conclusions and Future Work

In this study, an embedded multi-agent systems-managed wireless sensor network was presented. A novel architecture was proposed in which several agents are embedded in each sink node to manage clusters in a distributed fashion, and one agent is embedded in each sensor node. Active or query-based and passive or advertisement-based wireless sensor node types are compared. Two application-specific examples are also presented in this paper that illustrate the type of industrial WSN referenced in this paper. The first example, which is an example from a previous agent-based WSN, is an agent-based WSN for the oil and gas refining process. The second example is a novel embedded agent-based WSN for a tank storage facility, which requires the plug and play characteristics of the agent-embedded WSN.

This paper also presents a series of three experiments. The results of these experiments show that increasing the size of a cluster of sensor nodes increases the lost signal ratio and increases the power required for each sensor node, which coincides with the results of many previously performed experiments. The results also show that active or query-based wireless sensor nodes produce a lower lost signal ratio and require lower amounts of power than passive or advertisement-based wireless sensor nodes. Most importantly, the experiments show that dynamic clusters of wireless sensor nodes do not have a higher lost signal ratio or require more power than static WSN clusters. This is important as the dynamic characteristic is required for the WSN in the application-specific examples presented in this paper.

One of the questions that arises from this study is if there is a link between the lost signal ratio and power consumption. While this may be caused by the higher amount of time in the “transmission” state, further experimentation to determine if lowering the lost signal ratio lowers the power consumption of WSNs should be conducted. Another question that may be considered is whether the embedded agent architecture performs better than a similar cloud-based architecture. Further experimentation comparing embedded and cloud-based architectures for managing and controlling WSNs will need to be conducted.
